# Environmental Filtering Maintains Macroinvertebrate Diversity in the Upper Jinsha River

**DOI:** 10.1002/ece3.72323

**Published:** 2025-10-16

**Authors:** Xiaopeng Tang, Kunyu Shang, Lin Chen, Chunling Wang, Fubin Zhang, Pengcheng Lin

**Affiliations:** ^1^ College of Environmental Science and Engineering China West Normal University Nanchong China; ^2^ Institute of Hydrobiology Chinese Academy of Sciences Wuhan Hubei China; ^3^ Key Laboratory of Aquatic Biodiversity and Conservation of Chinese Academy of Sciences, Institute of Hydrobiology Chinese Academy of Sciences Wuhan China; ^4^ PowerChina Chengdu Engineering Corporation Limited Chengdu China

**Keywords:** biodiversity conservation, community assembly, environmental stressors, spatial processes

## Abstract

Elucidating the mechanisms that influence the assembly and maintenance of communities is a crucial objective in ecological research. However, the mechanisms underlying the maintenance of macroinvertebrate community structure in large rivers on the Tibetan Plateau are still not well understood. To this end, we systematically investigated the spatial distribution characteristics of community diversity and its maintenance mechanisms, focusing on the upper Jinsha River as our study region. During the period from November to December 2022, we collected a total of 126 macroinvertebrate species, representing 5 phyla, 7 classes, 14 orders, 53 families, and 96 genera. Our findings revealed notable variations in species composition among macroinvertebrate communities inhabiting the mainstem and its tributaries. Macroinvertebrate densities, biomass, and species richness were significantly higher in tributaries compared to the mainstem. Additionally, there were significant differences in the Margalef richness index, Pielou evenness index, Shannon diversity index, functional richness, and functional divergence when comparing the mainstem and tributary streams, whereas phylogenetic diversity showed no significant variations. Redundancy analysis demonstrated that the structure of macroinvertebrate communities was notably influenced by a combination of environmental and spatial variables, although the key factors varied among different water bodies. Furthermore, variance partitioning analysis indicated that deterministic processes predominantly shaped macroinvertebrate community assembly, while stochastic processes had a secondary influence. These findings enhance our understanding of macroinvertebrate community dynamics in high‐altitude river systems and provide a scientific basis for the conservation of riverine ecosystems and aquatic biodiversity in the upper Jinsha River.

## Graphical abstract

Macroinvertebrate community structure differed significantly among water bodies. Species and functional diversity are higher in tributaries than in mainstems, whereas phylogenetic diversity shows no significant spatial differences. The macroinvertebrate community in the upper Jinsha River are mainly deterministic processes.

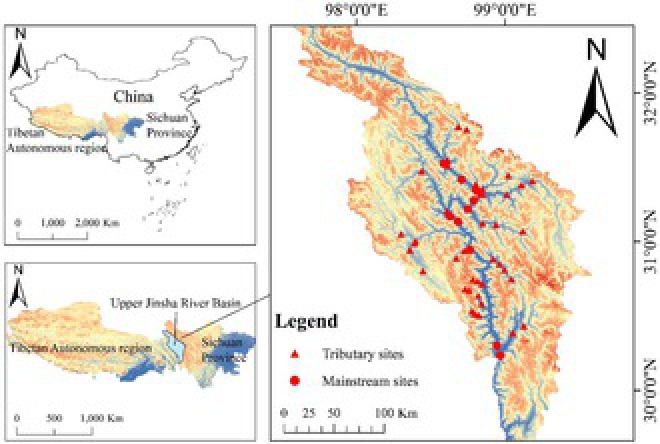

## 1 Introduction

A key focus of community ecology research is to assess species composition and elucidate the mechanisms underlying ecological community assembly (Cardinale et al. 2012). Understanding the mechanism of community assembly and maintenance is fundamental to the advancement of community ecology (Heino 2011). To this end, ecologists have formulated a variety of theoretical frameworks. Within Hutchinson's (1959) theoretical framework, trade‐offs in life‐history strategies (particularly in resource utilization patterns) serve as essential mechanisms that facilitate niche differentiation and thereby promote species coexistence (Silvertown 2004; Vandermeer 1972). Classical niche theory posits that natural communities arise from the interplay of environmental filtering and interspecific interactions among species (Colwell and Rangel 2009; Levine and HilleRisLambers 2009; Pulliam 2000). Ecological niche theory has traditionally been the dominant framework for explaining the factors that shape community composition and patterns of diversity (Levine and HilleRisLambers 2009; Loke and Chisholm 2023). However, recent studies have highlighted the limitations of ecological niche theory in forecasting changes in community structure (Loke and Chisholm 2023). Consequently, community neutrality theory has emerged as an alternative. The theory suggests that dispersal limitation is the predominant factor affecting species coexistence (Niu et al. 2009). However, this theory has been extensively challenged due to the assumption of ecological equivalence among species, which is inconsistent with empirical findings (Kraft et al. 2015). At present, a growing body of research is merging ecological niche theory with neutral theory to investigate the respective roles of stochastic and deterministic processes in the community (Ge et al. 2021; Li et al. 2020).

Determining the respective contribution of deterministic and stochastic processes in the mechanisms of community construction continues to pose a substantial challenge to contemporary research endeavors (Jiang et al. 2021). Environmental filtering considers environmental conditions as a “filter” that permits only species with specific traits to colonize and persist (Li, Chen, Jiang, et al. 2021). Consequently, adequate dispersal is crucial for species to adapt to diverse environmental conditions across various sites (Li, Heino, Chen, et al. 2021). Therefore, discerning the respective contributions of ecological niche and neutral processes to community assembly is pivotal for deepening our comprehension of the mechanisms underlying species coexistence (Liu, Zhou, et al. 2021).

Conventional research has predominantly concentrated on species diversity. The composition of biomes and their distribution patterns across time and space are described using a variety of indices based on species richness and abundance (Heino et al. 2017). Species diversity, which is based on taxonomic information, treats different species as equivalent units and is widely used in research (Baker et al. 2023). However, species display considerable variation in physiological, ecological, and functional characteristics (Ao et al. 2022; Liu, Zhang, et al. 2021). Hence, relying exclusively on species diversity does not adequately illustrate the crucial function of biodiversity in ecosystems (Li et al. 2020).

In recent years, functional and phylogenetic diversity has increasingly gained attention (Heino et al. 2015; Tilman 2001). Many researchers have advocated integrating species diversity with these two aspects in biodiversity research to conduct more comprehensive investigations (Li, Heino, Liu, et al. 2021; Li, Jiang, Wang, et al. 2019). Functional diversity pertains to the functional traits exhibited by all species within a particular community, as well as the degree of variation among these traits (Li et al. 2020). It emphasizes the variability of species functions within a community (Dai et al. 2024). Additionally, functional diversity includes information on species richness, community composition, and species traits (Ao et al. 2023; Calow 1987; Petchey and Gaston 2002). Therefore, it offers more comprehensive information than taxonomic diversity. Conversely, phylogenetic diversity takes into account the evolutionary relationships among the various species within a community (Heino et al. 2015). It reflects both the process of community assembly and the evolutionary history of species. Thus, integrating these three facets of diversity provides a more holistic comprehension of the mechanisms that govern the formation of community structure (Arnan et al. 2017). Currently, most relevant studies focus on ecosystems such as marine, estuarine, lakes, and small to medium‐sized rivers (Cai et al. 2019; He et al. 2020; Medeiros et al. 2021; Wan et al. 2024). Research on large rivers at high altitudes remains limited.

Our investigation was carried out in the upper Jinsha River, a section of the Yangtze River. Nevertheless, research on macroinvertebrates in this section remains relatively limited (Chi, Hu, et al. 2024; Chi et al. 2022). Macroinvertebrates constitute one of the most extensively distributed animal groups within aquatic environments (Boota et al. 2024a, 2024b; Jiang et al. 2013; Soomro et al. 2024, 2023). They occupy an intermediate position in the food web. Moreover, macroinvertebrates are pivotal in mediating energy flow and material cycling within these aquatic environments (Li et al. 2025). Most macroinvertebrates exhibit weak migratory capabilities and have limited ranges. In addition, they are highly diverse, and different taxa exhibit varying sensitivities to environmental change (Liu, Zhou, et al. 2021). Consequently, macroinvertebrates are commonly employed as bioindicators to assess the environmental conditions of rivers (Li et al. 2020). Recent studies on macroinvertebrates in the Jinsha River primarily examine community composition and ecological niche assessments (Chi, Hu, et al. 2024; Chi et al. 2022). Nevertheless, the mechanisms that govern community assembly remain poorly understood. Our research aims to: (1) investigate the spatial patterns of macroinvertebrate community diversity; (2) identify the principal environmental and spatial factors that influence macroinvertebrate communities; (3) elucidate the relative contributions of ecological niche processes and neutral processes to macroinvertebrate community assembly. We expect that our findings will offer valuable data support for the conservation of macroinvertebrates and ecosystems in the Jinsha River.

## 2 Materials and Methods

### 2.1 Study Area

**FIGURE 1:**
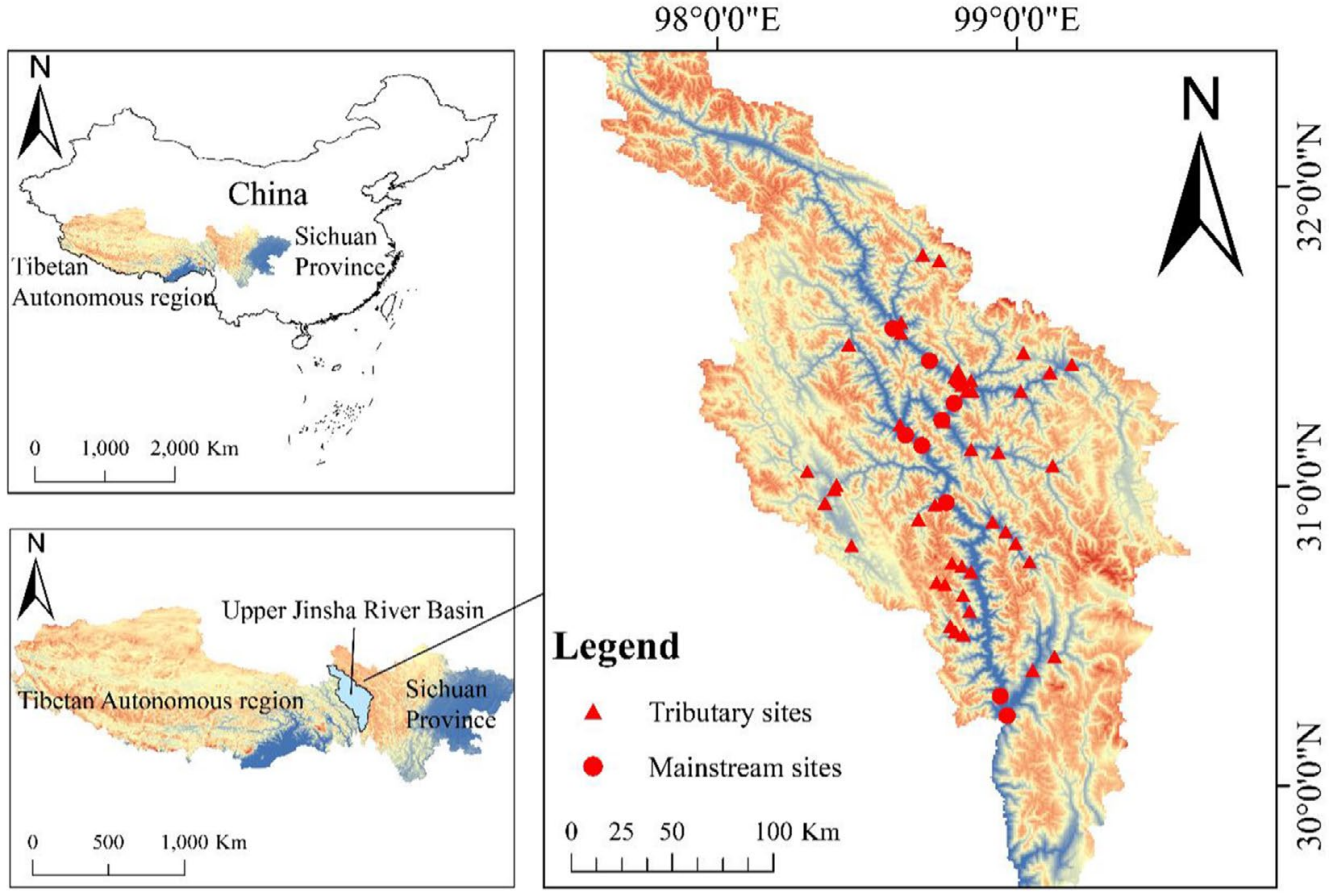
Sampling sites within the upper Jinsha River.

The Jinsha River, the upper section of the Yangtze River, crosses the Tibetan Plateau, the Yunnan‐Guizhou Plateau, and the Sichuan Basin in China. Our study region is situated in the upstream section of the Jinsha River (Figure 1). This river section originates at Zhimenda in Yushu City, Qinghai Province, traverses Sichuan Province and Tibet Autonomous Region, and extends to Shigu Town in Yunnan Province. The area extends over a total length of approximately 965 km and covers a watershed region of 2.6 × 10^5^ km^2^. The region is marked by complex geological conditions and diverse topography (Hu et al. 2022). It is characterized by high peaks and steep canyons (Chi, Hu, et al. 2024; Yao et al. 2023).

The Upper Jinsha River Basin exhibits remarkable three‐dimensional climatic characteristics and spatial heterogeneity. The region is characterized by a typical monsoon climate, with a rainy season from May to October, annual precipitation of approximately 600 mm, and an increasing trend along the longitudinal gradient of the river (from upstream to downstream) (Chi, Zheng, et al. 2024; Yuan et al. 2021). Temperature decreases from south to north, with an average annual temperature of about 5°C (Chi, Hu, et al. 2024). The special confluence of climatic characteristics, topography, and diverse ecosystems renders the upper Jinsha River a biodiversity hotspot (Hu et al. 2022; Zhang et al. 2020).

### 2.2 Macroinvertebrate Sampling

In this study, a systematic field survey was conducted in the upper Jinsha River basin from November to December 2022. Using standardized sampling methods, we established 55 sampling sites in the study area, comprising 10 sites in the mainstem and 45 sites in tributaries (spanning 13 major tributaries). The tributaries sampled included seven from the left bank: Sequ (SQ), Baiqu (BQ1), Dingqu (DQ1), Zengqu (ZQ1), Ouqu (OQ), Jiangqu (JQ), and Baqu (BQ2); and six from the right bank: Zangqu (ZQ2), Xiequ (XQ1), Minduhe (MDH), Dongqu (DQ2), Requ (RQ), and Xiqu (XQ2).

At every sampling location, three quantitative replicate samples were collected from a 100‐m section of the river. The collection was based on habitat characteristics, including substrate composition, water depth, and flow speed. Macroinvertebrates were gathered with a Surber sampler (30 × 30 cm, 500 μm mesh). Samples collected were sieve‐washed to extract the macroinvertebrates, which were then separated on a white dissecting tray. Thereafter, the picked samples were preserved in 50 mL specimen bottles containing a 75% ethanol solution. Species identification was conducted based on relevant literature (Brinkhurst et al. 1990; Liu et al. 1979; Morse et al. 1994; Zhou et al. 2003), with efforts to identify samples to the lowest taxonomic level.

### 2.3 Environmental Variables

Habitat indicators were concurrently measured at the sample site during the collection of macroinvertebrate samples. The sample site's geographical coordinates—latitude, longitude, and elevation—were recorded with a Garmin 60 s GPS. Stream width (Wid) was measured with a Leica rangefinder. Moreover, water temperature (WT), dissolved oxygen (DO), pH, conductivity (Cond), and oxidation–reduction potential (ORP) were evaluated with a YSI Pro Plus multiparameter water quality instrument. At each sampling site, triplicate measurements were obtained for each environmental parameter, with the mean value being utilized in subsequent analyses.

### 2.4 Spatial Variables

The principal coordinates of neighbor matrices (PCNM) method was utilized to quantify spatial variability among sample points (Borcard and Legendre 2002). This approach calculates and filters vectors with positive eigenvalues as spatial variables, based on the spatial coordinates of the sample points, to characterize stochastic processes. PCNM variables with larger eigenvalues (e.g., PCNM1) represent larger‐scale spatial processes, while those with smaller eigenvalues (e.g., PCNM34) indicate smaller‐scale spatial processes.

### 2.5 Data Analyses

#### 2.5.1 Diversity Indices

Initially, we characterized macroinvertebrate species diversity using the Margalef richness index, Pielou evenness index, Shannon diversity index, and Simpson diversity index (Hill 1973). Calculations for these indices were conducted utilizing the “vegan” package (Oksanen et al. 2024).

Subsequently, we employed phylogenetic diversity indices, including taxonomic diversity (Delta), taxonomic dissimilarity (Delta*), average taxonomic dissimilarity (Delta+), and variation in taxonomic dissimilarity (Lambda+) (Heino et al. 2015). These indices were derived from the Linnean taxonomic trees utilizing the “taxondive” and “taxa2dist” functions within the “vegan” package (Oksanen et al. 2024).

Thirdly, we selected 10 macroinvertebrate traits to assess functional diversity based on local habitat conditions and taxon characteristics (Table S1). Trait selection was informed by relevant literature (Poff et al. 2006). We quantified functional diversity using functional richness (FRic), functional evenness (FEve), functional divergence (FDiv), and functional dispersion (FDis). These metrics were calculated using the “dbFD” function within the “FD” package.

#### 2.5.2 Relationships Between Macroinvertebrate Community and Ecological Drivers

In this study, we used one‐way ANOVA to systematically assess the variability of environmental variables, macroinvertebrate densities, biomass, and diversity indices in the upper Jinsha River mainstem and its left‐ and right‐bank tributaries. To improve data normality, biological data (density, biomass, diversity indices) and environmental variables (except pH) were log(*x* + 1) transformed. Additionally, principal coordinates analysis (PCoA) and permutational analysis of variance (PERMANOVA) were utilized to evaluate differences in macroinvertebrate communities across various water bodies.

A constrained ordination approach was employed to analyze the associations among environmental factors, spatial variables, and the structure of the macroinvertebrate community. Initially, detrended correspondence analysis (DCA) was performed on macroinvertebrate community data from the upper Jinsha River to assess the length of the longest gradient. This result indicated that a linear model was suitable for analyzing community‐environment relationships, as the gradient length was less than three standard deviations. Consequently, redundancy analysis (RDA) was chosen for this study. To enhance the robustness of the analyses, Hellinger transformations were applied to density data. To ensure data normality and homogeneity of variances, log(*x* + 1) transformations were utilized for the environmental data, with the exception of pH. Environmental parameters exhibiting high correlations (*r* > 0.80) or VIF exceeding 20 were excluded from the analysis. Key environmental and spatial factors were determined through forward selection coupled with Monte Carlo permutation tests.

Finally, variance partitioning analysis (VPA) was employed to explore the importance of environmental filtration and spatial effects on variation in community structure. A partial redundancy analysis was conducted to assess the overall variation in macroinvertebrate community composition. This approach decomposed the variation into four components: one explained solely by environmental factors, one by spatial factors, one by the interaction of both environmental and spatial factors, and an unexplained component.

One‐way ANOVA was performed using SPSS 22.0. The remaining statistical analyses, including PERMANOVA, PCoA, PCNM, RDA, and VPA, were conducted in the R 4.4.0 environment (R Core Team 2024). Community ecology analyses were performed using the vegan package (Oksanen et al. 2024), which is available from the CRAN repository (https://CRAN.R‐project.org/package=vegan).

## 3 Results

### 3.1 Environmental Features

**FIGURE 2:**
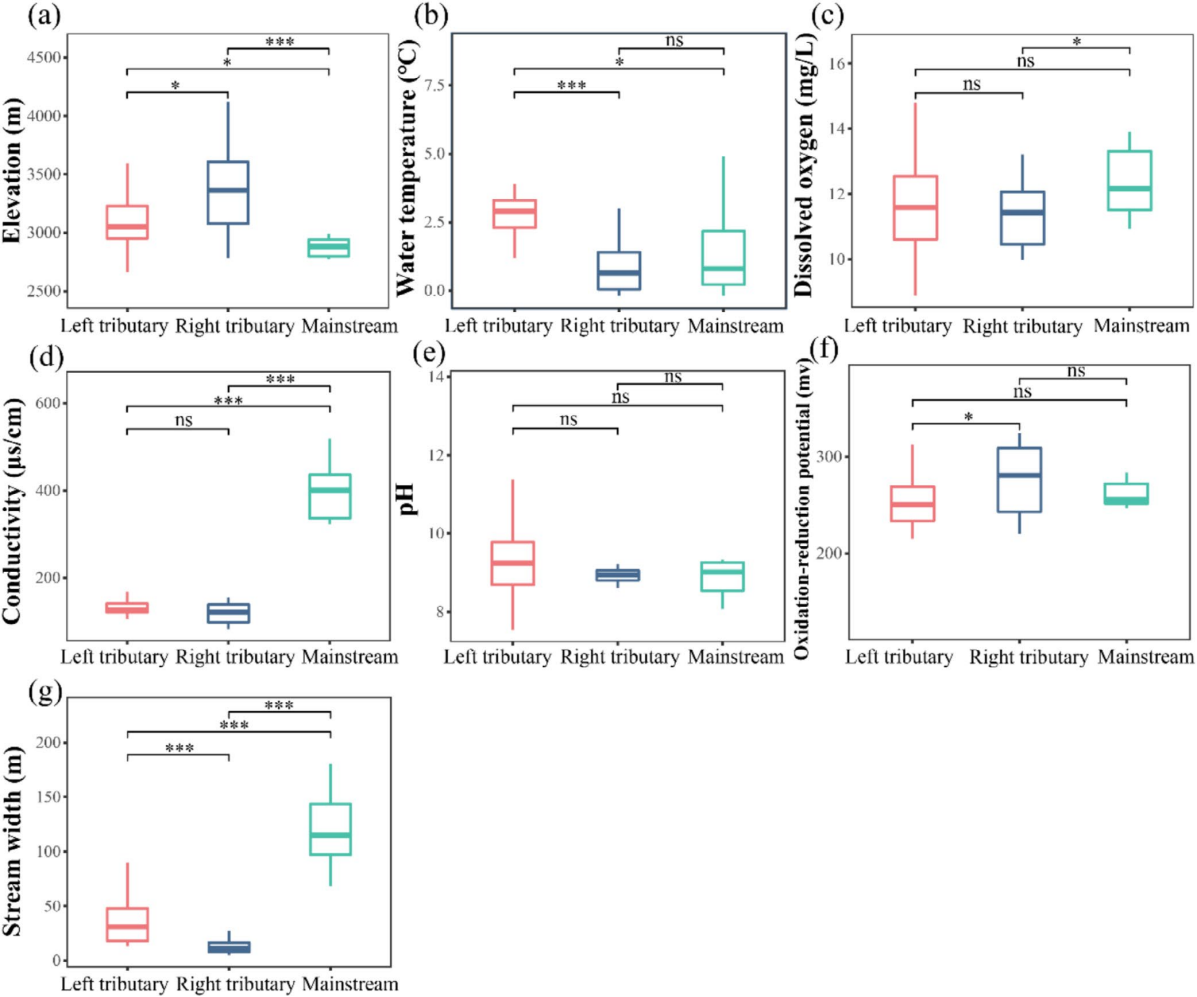
Boxplots of environmental factors in the upper Jinsha River. (a) Elevation. (b) Water temperature. (c) Dissolved oxygen. (d) Conductivity. (e) pH. (f) Oxidation‐reduction potential. (g) Stream width. The central horizontal line within the box represents the median value. The upper and lower borders of the box represent the upper and lower quartiles of the dataset, with whiskers extending to 1.5 times the interquartile range. Asterisks indicate statistically significant differences (*p < 0.05, ***p < 0.001). Non‐significant results are denoted as “ns.”

The upper Jinsha River exhibited distinct environmental characteristics across various water bodies (Figure 2). Significant differences were observed in most environmental factors between the mainstem and tributaries. Specifically, both left (median = 3053, IQR = 299.00) and right bank tributaries (median = 3364, IQR = 596.50) had significantly higher elevations than the mainstem (median = 2883, IQR = 247.75). Water temperatures were significantly higher in left bank tributaries (median = 2.9, IQR = 1.60) compared to right bank tributaries (median = 0.7, IQR = 1.85) and the mainstem (median = 0.8, IQR = 2.60). Additionally, conductivity and stream width exhibited significant variations between the mainstem and tributaries. The mainstem showed higher median conductivity (391.3 μS/cm, IQR = 116.20) and stream width (115 m, IQR = 60.75) compared to tributaries. Among tributaries, left tributaries had median conductivity of 126.6 μS/cm (IQR = 20.60) and stream width of 31 m (IQR = 30.00), while right tributaries showed lower values (conductivity: 122.0 μS/cm, IQR = 42.58; stream width: 11 m, IQR = 13.50). However, dissolved oxygen levels did not differ significantly between the mainstem (median = 12.16, IQR = 2.17) and left bank tributaries (median = 11.59, IQR = 2.31). Notably, pH values exhibited minimal variation between the mainstem (median = 9.02, IQR = 1.41) and tributaries, with left tributaries showing median pH of 9.24 (IQR = 1.20) and right tributaries 8.94 (IQR = 0.27).

### 3.2 Macroinvertebrate Community Structure

**FIGURE 3:**
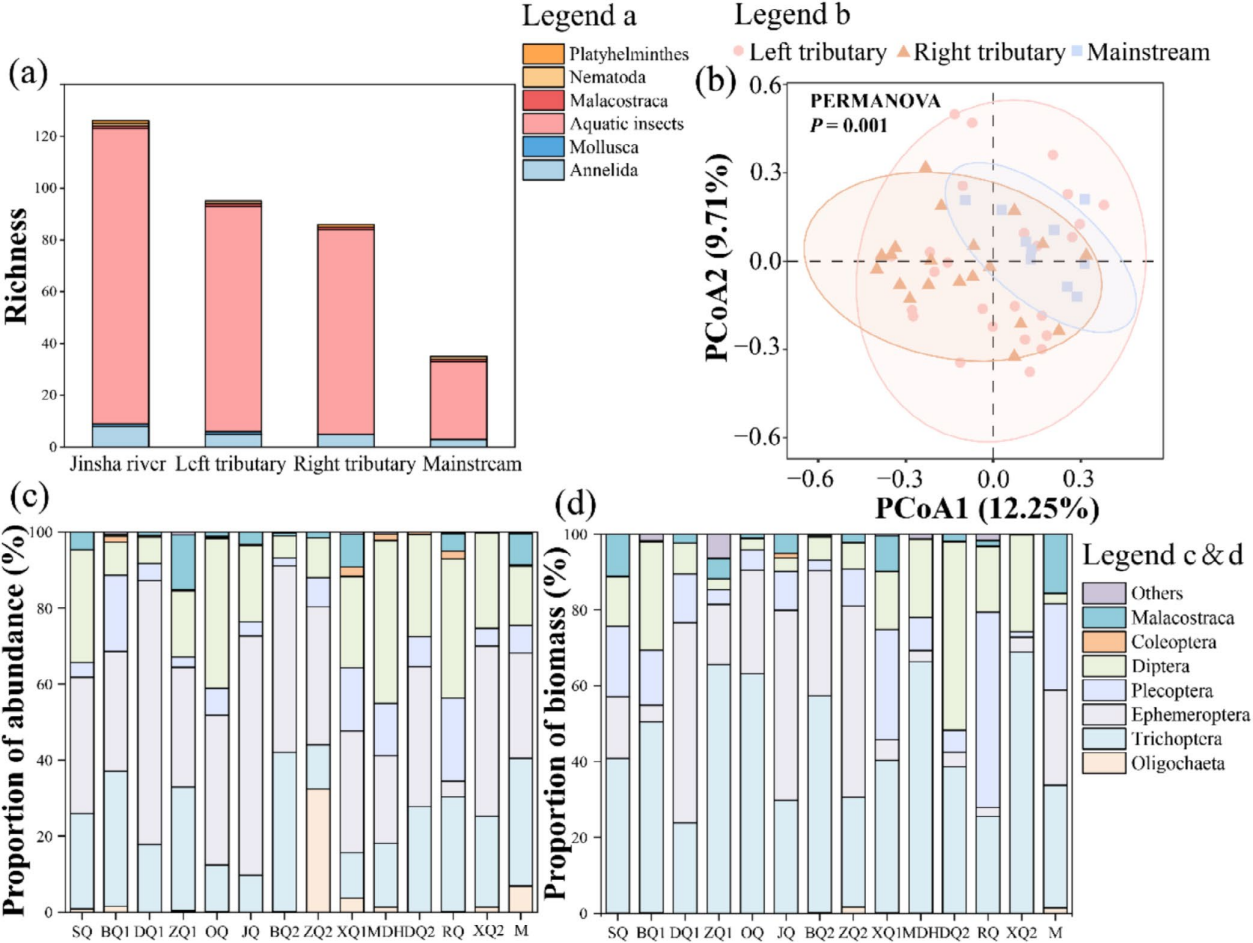
Macroinvertebrate community characteristics across different water bodies. (a) Species richness in the basin, tributaries, and mainstem. (b) Principal coordinate analysis (PCoA) of community composition between mainstem and tributary streams. (c) Mean abundance and (d) mean biomass of macroinvertebrates in the study area. Stream abbreviations: SQ (Sequ), BQ1 (Baiqu), DQ1 (Dingqu), ZQ1 (Zengqu), OQ (Ouqu), JQ (Jiangqu), BQ2 (Baqu), ZQ2 (Zangqu), XQ1 (Xiequ), MDH (Minduhe), DQ2 (Dongqu), RQ (Requ), XQ2 (Xiqu), and M (Mainstream).

During this study, a total of 126 taxa of macroinvertebrates were identified. These were represented in 5 phyla, 7 classes, 14 orders, 53 families, and 96 genera. Among these, 124 species were detected in the tributaries, while 35 were found in the mainstem. Aquatic insects constituted the dominant group, with 114 species representing 90.48% of the total species (Figure 3a). Additionally, eight species of Annelida (6.35%) were identified, along with one species each of Mollusca, Malacostraca, Nematoda, and Platyhelminthes (0.79%).

The mean density and biomass of macroinvertebrates across the basin were 872.69 ind/m^2^ and 12.49 g/m^2^, respectively. One‐way ANOVA indicated notable differences in density and biomass of macroinvertebrate communities between tributaries and the mainstem (*p* < 0.05) (Table 1). Specifically, mean density was higher in tributaries (757.59 ind/m^2^) than in the mainstem (96.33 ind/m^2^) (Figure 3c). Similarly, mean biomass was higher in tributaries (10.89 g/m^2^) than in the mainstem (0.58 g/m^2^) (Figure 3d).

**TABLE 1:** Results of one‐way ANOVA for macroinvertebrate density and biomass in different water bodies.

	Left tributary	Right tributary	Mainstream	*F*	*p*
Density	603.19 ± 747.50	911.99 ± 920.70	12.04 ± 11.82	17.057	0.001
Biomass	8.05 ± 11.54	13.73 ± 20.14	0.07 ± 0.08	7.816	0.014

PCoA analysis revealed significant variations in macroinvertebrate communities between mainstem and tributary streams (Figure 3b). PCoA1 explained 12.25% of the variation in benthic community structure, while PCoA2 accounted for 9.71%. Furthermore, PERMANOVA analysis also revealed notable variations in the macroinvertebrate community composition across different water bodies (*p* < 0.05) (Table 2).

**TABLE 2:** Results of PERMANOVA analysis for macroinvertebrate community structure in different water bodies of the upper Jinsha River.

	df	Sum of squares	*R* ^2^	*F*	*p*
Water bodies	2	1.974	0.094	2.692	0.001
Residual	52	19.064	0.906		
Total	54	21.038	1		

### 3.3 Macroinvertebrate Diversity

**FIGURE 4:**
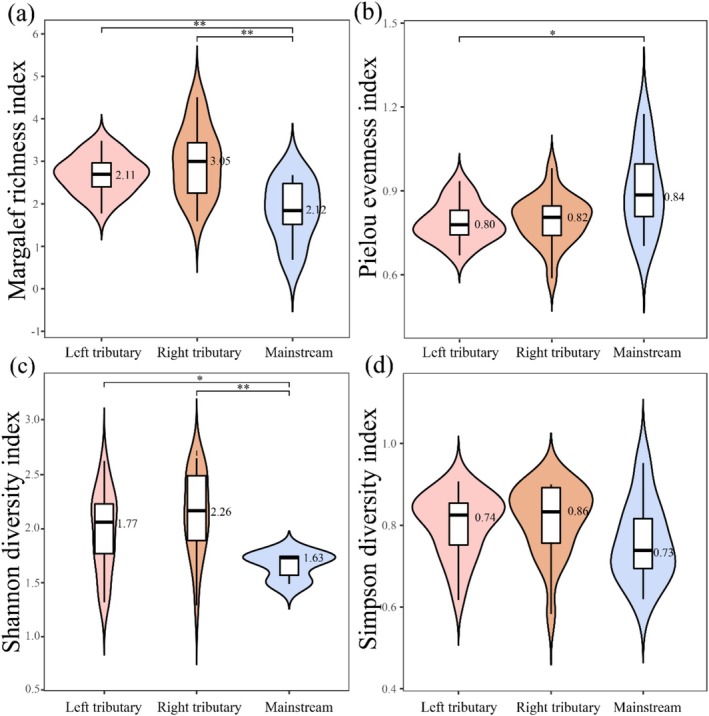
Macroinvertebrate species diversity patterns in the upper Jinsha River basin: (a) Margalef richness index. (b) Pielou evenness index. (c) Shannon diversity index. (d) Simpson diversity index across different water body types (mainstem vs. tributaries). The black horizontal line within the box denotes the median biodiversity index. The top extension line indicates the data range within 1.5 times the interquartile range. The upper and lower borders of the white box represent the upper and lower quartiles of the dataset. Asterisks mark significant differences (*p < 0.05, **p < 0.01).

One‐way ANOVA revealed significant spatial differences in macroinvertebrate community diversity across the upper Jinsha River (Figure 4). The Margalef richness index was highest in the right bank tributaries (median = 3.05, interquartile range (IQR) = 1.19), significantly exceeding values in the left bank tributaries (median = 2.11, IQR = 0.85) and the mainstem (median = 2.12, IQR = 1.76) (*p* < 0.01). Similarly, the Shannon diversity index was markedly elevated in the right bank tributaries (median = 2.26, IQR = 0.61) compared to the left bank tributaries (median = 1.77, IQR = 0.59) and the mainstem (median = 1.63, IQR = 0.64), with greater variability observed in the tributaries. In contrast, the mainstem exhibited the highest Pielou evenness index (median = 0.84, IQR = 0.28), surpassing both the left (median = 0.80, IQR = 0.15) and right (median = 0.82, IQR = 0.11) tributaries. No significant spatial differences were detected for the Simpson index (right tributaries: median = 0.86, IQR = 0.14; left tributaries: median = 0.74, IQR = 0.12; mainstem: median = 0.73, IQR = 0.15). Collectively, these results indicate that the right bank tributaries support higher species richness and diversity, whereas the mainstem maintains greater evenness.

**FIGURE 5:**
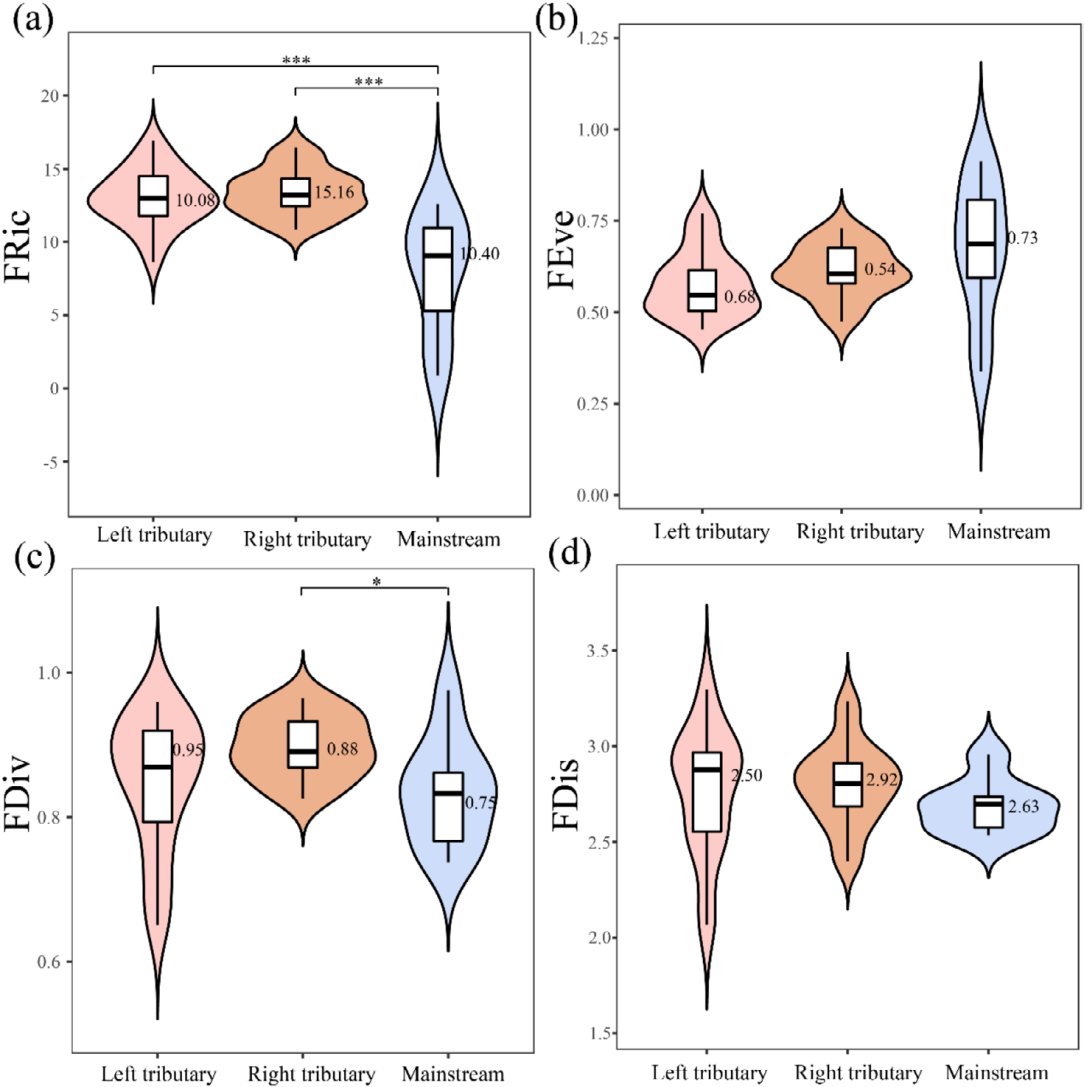
Macroinvertebrate functional diversity patterns in the upper Jinsha River basin: (a) FRic (functional richness). (b) FEve (functional evenness). (c) FDiv (functional divergence). (d) FDis (functional dispersion) across different water body types (mainstem vs. tributaries). The black horizontal line within the box denotes the median biodiversity index. The top extension line indicates the data range within 1.5 times the interquartile range. The upper and lower borders of the white box represent the upper and lower quartiles of the dataset. Asterisks mark significant differences (*p < 0.05, ***p < 0.001).

Functional diversity indices exhibited distinct spatial patterns across the river system (Figure 5). The right bank tributaries demonstrated the highest functional richness (FRic: median = 15.16, IQR = 1.96) and functional dispersion (FDis: median = 2.92, IQR = 0.27), but the lowest functional evenness (FEve: median = 0.54, IQR = 0.09). In contrast, the mainstem showed the highest FEve (median = 0.73, IQR = 0.40) but the lowest functional divergence (FDiv: median = 0.75, IQR = 0.12), while left bank tributaries exhibited the highest FDiv (median = 0.95, IQR = 0.14). Significant spatial variations were observed for FRic and FDiv (*p* < 0.05), with tributaries generally showing higher values than the mainstem. The mainstem displayed particularly strong heterogeneity in FRic (IQR = 9.25) and FDis (IQR = 0.74). Across all sites, index ranges were: FRic (0.91–16.92), FEve (0.34–0.91), FDiv (0.50–0.97), and FDis (0.99–3.30), with respective means of 11.99, 0.61, 0.85, and 2.70.

**FIGURE 6:**
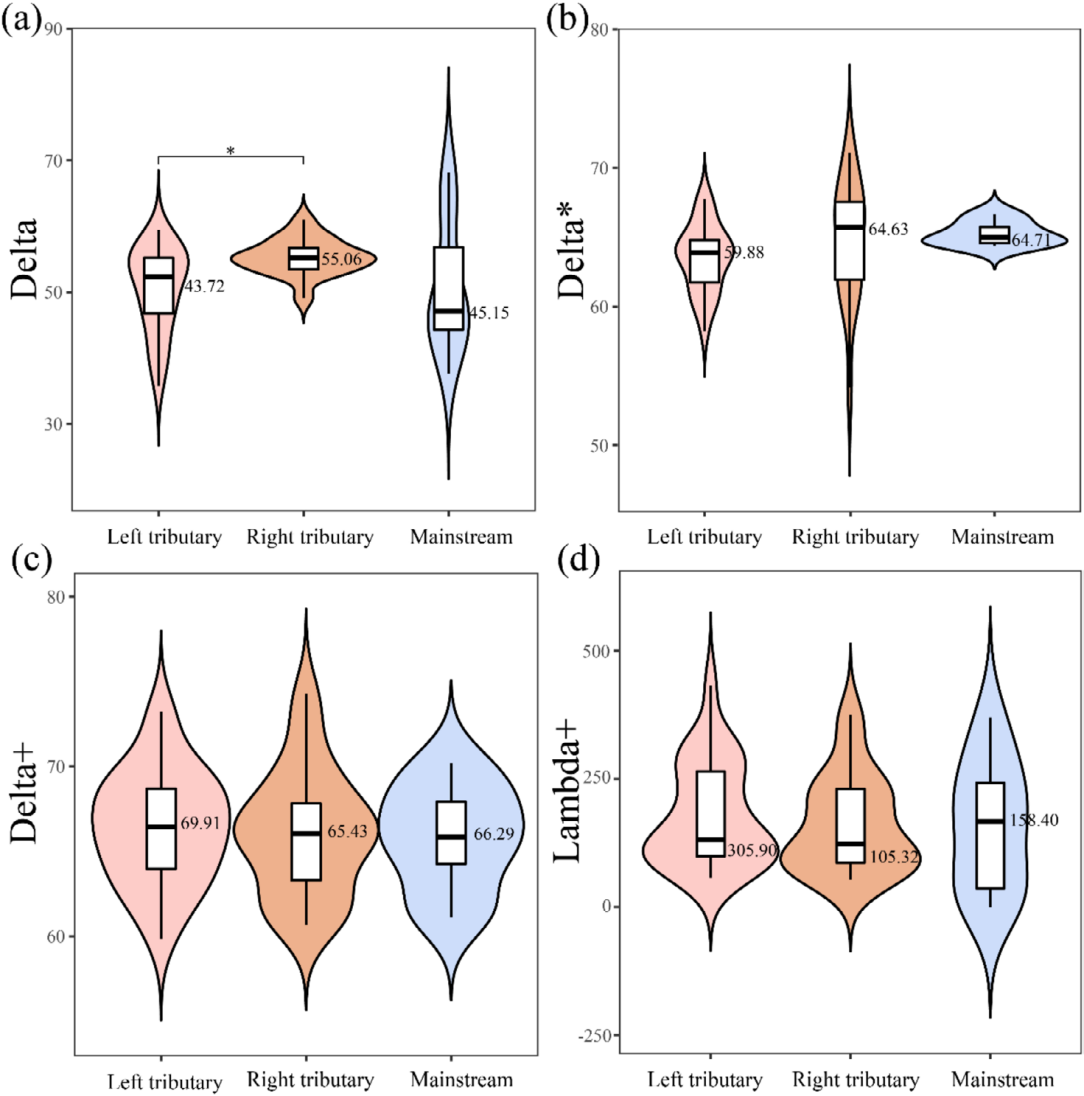
Macroinvertebrate phylogenetic diversity patterns in the upper Jinsha River basin: (a) Delta (taxonomic diversity). (b) Delta* (taxonomic dissimilarity). (c) Delta+ (average taxonomic dissimilarity). (d) Lambda+ (variation in taxonomic dissimilarity) across different water body types (mainstem vs. tributaries). The black horizontal line within the box denotes the median biodiversity index. The top extension line indicates the data range within 1.5 times the interquartile range. The upper and lower borders of the white box represent the upper and lower quartiles of the dataset. Asterisks mark significant differences (*p < 0.05).

In comparison to functional diversity, there were no notable variations in the phylogenetic diversity index between the mainstem and tributary (Figure 6). Nevertheless, significant differences in Delta were observed between left and right bank tributaries. While no significant differences were found between mainstem and tributary streams for Delta*, Delta+, and Lambda+, right bank tributaries showed the highest Delta values (median = 55.06, IQR = 3.52), and the mainstem displayed the highest Delta* (median = 64.71, IQR = 7.33). Left bank tributaries outperformed other sections in both Delta+ (median = 69.91, IQR = 5.45) and Lambda+ (median = 305.90, IQR = 165.93). Notably, the mainstem exhibited the greatest variability in Delta (IQR = 15.46) and Lambda+ (IQR = 229.3), indicating strong spatial heterogeneity. In contrast, right bank tributaries showed relatively stable phylogenetic diversity (Delta IQR = 3.52; Delta+ IQR = 5.36). These results demonstrate that while tributaries collectively maintained higher phylogenetic diversity than the mainstem, significant differences emerged between left and right bank tributaries (*p* < 0.05).

### 3.4 Key Drivers of Communities

**FIGURE 7:**
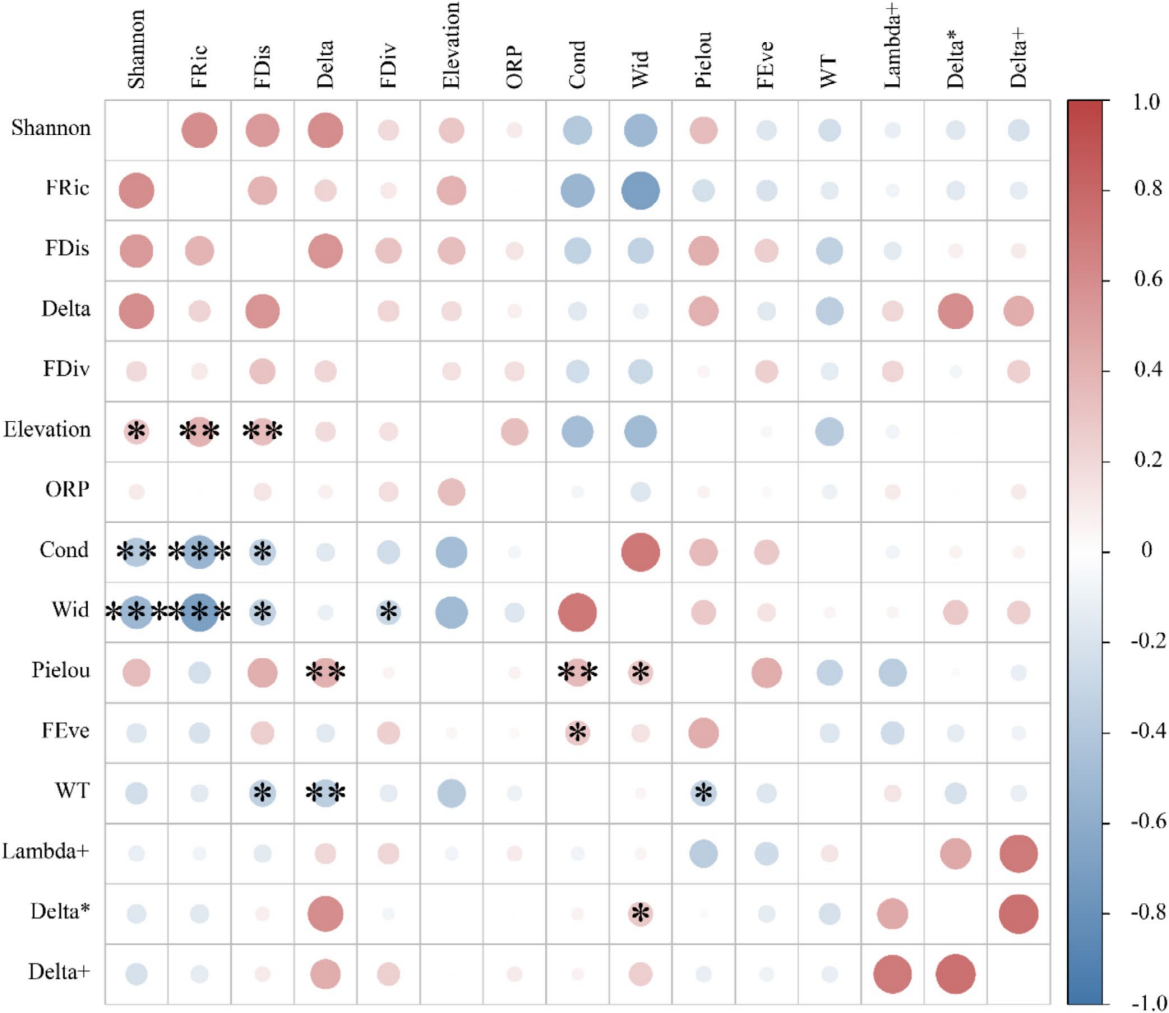
Relationship between macroinvertebrate diversity indices and environmental parameters. The figure presents only statistically significant correlations (*p* < 0.05) between biodiversity indices and environmental variables; non‐significant correlations are not shown. Cond, conductivity; Delta*, taxonomic dissimilarity; Delta, taxonomic diversity; Delta+, average taxonomic dissimilarity; FDis, functional dispersion; FDiv, functional divergence; FEve, functional evenness; FRic, functional richness; Lambda+, variation in taxonomic dissimilarity; ORP, oxidation–reduction potential; Wid, stream width; WT, water temperature. (*p < 0.05, **p < 0.01, and ***p < 0.001).

Through correlation analyses, it was found that the Pielou evenness index had a significant positive association with both conductivity and stream width. Instead, the Shannon diversity index exhibited a marked negative relationship with these variables (Figure 7). Moreover, a significant positive relationship was detected between FRic and FDis and elevation. By contrast, a significant negative association was found between these variables and both conductivity and stream width. FDiv exhibited a negative correlation with stream width, whereas FEve showed a positive correlation with conductivity. In terms of the phylogenetic diversity, Delta was negatively correlated with water temperature. In contrast, Delta* showed a positive correlation with stream width.

**FIGURE 8:**
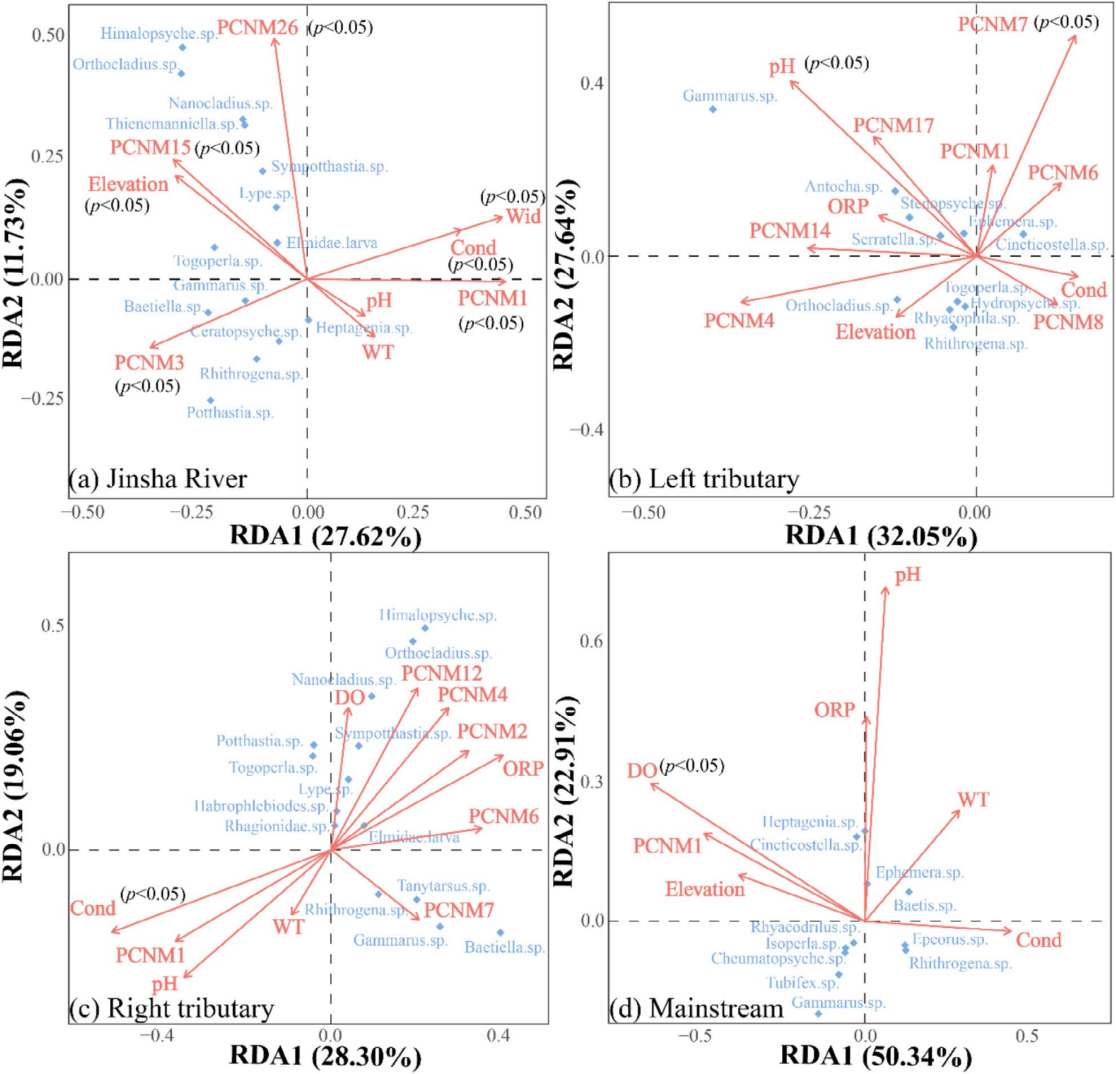
Redundancy analysis for macroinvertebrate community structure with environmental and spatial parameters across major water bodies of the study area. (a) Jinsha River. (b) Left tributary. (c) Right tributary. (d) Mainstream. Cond, conductivity; DO, dissolved oxygen; ORP, oxidation–reduction potential; Wid, stream width; WT, water temperature.

Redundancy analysis demonstrated that the primary environmental and spatial variables having an impact on the macroinvertebrate communities were distinct across different water bodies (Figure 8). Specifically, the basin‐wide key factors comprised elevation, conductivity, stream width, and the spatial components PCNM1, PCNM3, PCNM15, and PCNM26. For left bank tributaries, pH and PCNM7 were the primary factors. Right bank tributaries were predominantly affected by conductivity. In addition, dissolved oxygen was identified as a key factor for mainstem streams.

**FIGURE 9:**
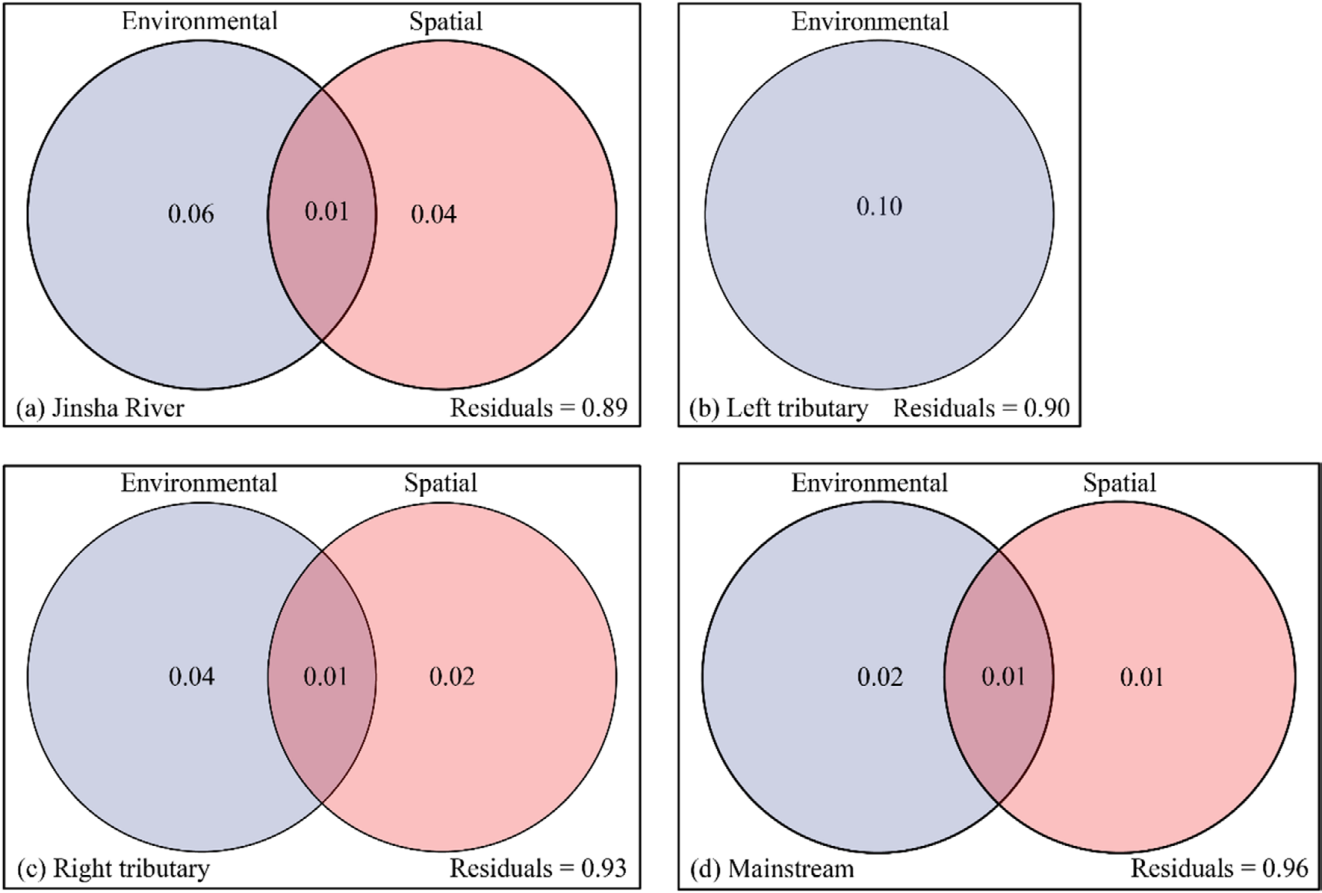
The respective contributions of environmental and spatial parameters in affecting community dynamics. (a) Jinsha River. (b) Left tributary. (c) Right tributary. (d) Mainstream.

VPA revealed that the assembly mechanisms of macroinvertebrate communities differed across various water bodies (Figure 9). At the basin‐wide scale, environmental and spatial variables jointly explained 10% of the variation in community structure, with environmental variables accounting for 6% and spatial factors for 4%. In left bank tributaries, environmental factors alone explained 10%. In contrast, in right bank tributaries, environmental and spatial variables collectively accounted for 6%, with environmental factors contributing 4%, spatial factors 2%, and a shared contribution of 1%. For mainstem streams, environmental and spatial variables jointly accounted for 3%, with environmental factors contributing 2%, spatial factors 1%, and a shared contribution of 1%. In general, it was discovered that both environmental and spatial factors had an impact on the formation of macroinvertebrate communities. However, environmental factors had a stronger impact than spatial variables.

## 4 Discussion

### 4.1 Community Structure and Diversity

The upper section of the Jinsha River is situated within the high mountain valley area of the Sichuan–Tibet–Yunnan region, characterized by turbulent waters and steep slopes along both banks (Yao et al. 2023). The complex topography and unique climatic conditions of this region provide an ideal habitat for a diverse range of aquatic taxa (Hu et al. 2022). However, macroinvertebrate surveys in this region have been relatively limited. Previous studies have predominantly focused on a few river sections or tributaries. As a result, this hinders the ability to offer a comprehensive and objective representation of macroinvertebrate diversity across the region. The present survey, which covered the mainstem and its 13 tributaries, represents one of the most comprehensive assessments of macroinvertebrates in the upper Jinsha River thus far.

During the investigation, we recorded a total of 126 macroinvertebrate species. This number is significantly higher than the 84 species reported by Chi, Zheng, et al. (2024). The differences may be attributed to variations in sampling seasons and the placement of sampling sites. In this region, macroinvertebrates are predominantly composed of Arthropoda, with aquatic insects being the dominant taxa. In contrast, the number of species of Mollusca, Annelida, Nematoda, and Platyhelminthes is relatively low. Aquatic insects have short life histories and can adapt to extreme plateau environments (Chi et al. 2022). The upper Jinsha River has been subjected to minimal human activity, thereby preserving its pristine condition (Yi et al. 2023). Consequently, the macroinvertebrate communities at the sampled sites were primarily dominated by sensitive species from the EPT taxa (Ephemeroptera, Plecoptera, and Trichoptera), as well as other aquatic insects. This explains the tendency of aquatic insects to predominate in macroinvertebrate communities within sandy and high‐elevation streams.

It is noteworthy that the spatial distribution of macroinvertebrate taxa in the upper Jinsha River exhibits considerable heterogeneity. EPT taxa, which are more sensitive to environmental changes (Hu et al. 2022), are predominantly found in tributary reaches. This may be attributed to the higher habitat heterogeneity in tributaries, where the substrate is primarily composed of sand and gravel. Such substrates are rich in dissolved oxygen and provide a suitable environment for the survival of EPT taxa (Bai et al. 2024). In contrast, mainstem reaches are characterized by greater depth, fewer rocks, wider channels, and slower flow rates. These characteristics exert a more pronounced influence on the composition of macroinvertebrate communities. This, to some extent, accounts for the differences in EPT taxa between the mainstem and tributaries. This survey identified *Physa* sp. of the Basommatophora as the mollusks present in the upper Jinsha River. This finding aligns with previous research (Chi et al. 2022). Based on the current survey data, mollusks in high‐altitude rivers are predominantly represented by the Basommatophora (Li et al. 2024; Ma et al. 2019). This indicates that this group of mollusks can adapt to extremely cold climates and survive in high‐altitude and alpine regions (Chi et al. 2022).

Spatial variations in community structure can result in differences in species diversity. This research found that the diversity of macroinvertebrates exhibited some variation between the mainstem and tributaries. Notably, the tributaries of this area exhibited higher levels of diversity. Prior research has indicated that more stable substrates are associated with increased habitat heterogeneity, thereby promoting greater biodiversity (Li et al. 2020). Compared to the mainstem, sampling sites of tributaries are characterized by higher elevations, more stable substrates, and greater habitat heterogeneity (Liu et al. 2016). Moreover, mainstem streams generally possess a simpler stream network structure, exhibit higher connectivity, and experience more pronounced anthropogenic disturbances relative to tributaries (Bai et al. 2024). These factors result in a decrease in species abundance and diversity within the community (Li, Jiang, Wang, et al. 2019). As a result, the higher species diversity observed in the tributaries, in comparison to the mainstem, can be attributed to these factors.

This study systematically assessed the species composition and diversity characteristics of macroinvertebrates in the upper Jinsha River. The basin exhibited high macroinvertebrate diversity with significant spatial heterogeneity, primarily driven by natural geographic heterogeneity and differences in species life history strategies. This research enriches the ecological data for macroinvertebrates in Tibetan Plateau rivers and provides a scientific basis for developing a biodiversity monitoring system and conservation strategies in the Jinsha River Basin.

### 4.2 Maintenance Mechanisms of Macroinvertebrate Diversity

Ecological processes include both deterministic and stochastic factors, which together drive the assembly of biological communities (Heino 2013). The interplay between these processes has a significant impact on species aggregation within communities (Lei et al. 2021). VPA analysis revealed that the structure of macroinvertebrate communities in the upper Jinsha River is shaped by both environmental and spatial parameters. This implies that community assemblages are influenced by both environmental filtering (ecological niche processes) and dispersal (neutral processes) (Heino 2013). Among these factors, environmental factors exerted a greater influence in explaining community variation. The findings of this study align with the majority of research on macroinvertebrate community aggregation (Dong et al. 2024; Li et al. 2020; Lin et al. 2024). During community assembly, environmental filtering often exerts a dominant influence in regions characterized by high environmental heterogeneity (Firmiano et al. 2021). The upper Jinsha River is distinguished by numerous tributaries, a complex river network, and variable hydrological conditions. This region supports a diverse macroinvertebrate community and exhibits high environmental heterogeneity (Chi et al. 2022). Macroinvertebrates in mountain rivers exhibit strong adaptations to environmental conditions, including rapids and low temperatures (Li et al. 2025). They are also extremely sensitive and vulnerable to environmental changes. Additionally, macroinvertebrate communities in mountain rivers are highly diverse and exhibit unique ecological and physiological characteristics (Sun et al. 2024). These include weaker mobility and varying sensitivities of different species to environmental changes. Consequently, the coupling between macroinvertebrate communities and environmental conditions is very strong. This renders environmental filtering a predominant factor in the macroinvertebrate community assembly in this region.

Environmental parameters are crucial in the environmental filtering process, significantly affecting the survival and growth of species (Fu et al. 2024). The structural characteristics of macroinvertebrate communities are closely associated with environmental changes. These organisms adjust their ecological adaptations to maintain the dynamic balance of ecosystems (Atristain et al. 2024). In this study region, elevation, conductivity, and stream width emerged as the principal environmental factors influencing macroinvertebrate communities. Among these, elevation, acting as an integrative factor, influences water temperature and light intensity (Ao et al. 2022). This, in turn, leads to changes in the species composition of macroinvertebrates. In this study, the investigation area covered a broad elevation range from 2458 to 4120 m. This notable elevational gradient is a major driver of the variations in the structure and diversity of macroinvertebrate communities. In this study region, particularly in its tributaries at higher elevations, a significant increase in the density and biomass of aquatic insects was observed. Conversely, specifically along the mainstem at lower elevations, a significant decline in the density and biomass of macroinvertebrates was noted. This partially explains the significant differences in macroinvertebrate densities and biomass observed between the tributaries and mainstem in the upper Jinsha River.

Conductivity is an essential environmental variable shaping the distribution of macroinvertebrate communities (Fu et al. 2024). It indicates the concentration of dissolved ions in the water column. Conductivity is positively correlated with the level of anthropogenic disturbance. It serves as a crucial indicator of river water quality degradation (Chen et al. 2016). Furthermore, conductivity is a key indicator of dissolved ion concentrations in the water column (Qin et al. 2023). Elevated conductivity has been shown to significantly increase ion concentrations, disrupting osmotic pressure homeostasis in aquatic macroinvertebrates (Zhang et al. 2023). This physiological stress can significantly impact macroinvertebrate communities, potentially altering species composition and community structure. Our results reveal a negative correlation between conductivity and macroinvertebrate communities. RDA analysis confirms this finding.

River width is an important driver affecting the formation of the macroinvertebrate communities (Jiang et al. 2013). It provides an indication of the river level to some extent (Tonkin et al. 2018). As river levels fluctuate, the local riverine environment undergoes a series of changes. Correspondingly, the species composition of the community is significantly altered (Li, Wang, Meng, et al. 2019). In the tributaries, the river channels are narrower and exhibit faster flow velocities, providing suitable habitats for insects such as EPT taxa. In contrast, in the mainstem, the confluence of tributaries at different elevations leads to a wider channel and a reduction in dissolved oxygen concentrations. This results in an increased abundance of small‐bodied aquatic oligochaetes and Chironomidae larvae. Thus, the findings of this study demonstrate that river width significantly affects the composition of macroinvertebrate communities.

At the same time, the role of spatial parameters in shaping communities was not overlooked. This indicates that dispersal‐related processes are also crucial in shaping the macroinvertebrate community assembly (Li, Wang, Liu, et al. 2019). Spatial effects on community assembly arise not only from dispersal limitations but can also result from mass effects (Heino et al. 2017). However, methods like ordination analysis and variance partitioning analysis struggle to differentiate between the effects of dispersal limitation and mass effects (Heino 2013). Typically, spatial effects are more pronounced in smaller areas or areas with higher connectivity. This suggests that high diffusivity (i.e., mass effect) significantly influences community aggregation (Li et al. 2020; Liu et al. 2024). Conversely, in larger spatial extents characterized by complex topography, geomorphology, and river network structure, dispersal limitation tends to dominate within the watershed (Li, Heino, Chen, et al. 2021). The upper Jinsha River encompasses an area of 2.6 × 10^5^ km^2^, exhibiting characteristics of high mountains, deep valleys, and steep canyons (Hu et al. 2022). Complex geologic environments and variable topography restrict species movement and exchange (Li, Heino, et al. 2023). Thus, dispersal constraints may be the primary factor driving the aggregation of macroinvertebrate communities in the upper Jinsha River. Notably, the variance partitioning analysis used in this study fails to account for all the variations in the macroinvertebrate community. This indicates that other variables, not considered in the present research, for instance, biotic interactions (e.g., competition, predation), regional extinctions, and further processes, may also play a significant role (Heino et al. 2017). Future research should encompass a wider array of ecological processes. Furthermore, diverse models and methods should be employed to more comprehensively elucidate the mechanisms that maintain macroinvertebrate communities in the upper Jinsha River.

This study elucidates the mechanisms underlying macroinvertebrate community assembly in the upper Jinsha River. We found that environmental filtering and spatial processes jointly drive community assembly, with environmental factors playing a particularly dominant role. This confirms that environmental filtering mechanisms are key factors shaping macroinvertebrate community structure in highland river ecosystems. Therefore, biodiversity conservation strategies in the Jinsha River basin should prioritize the regulation of key environmental factors.

## 5 Conclusion

In this study, we systematically assessed the species composition and diversity of macroinvertebrates in the upper Jinsha River and elucidated the key mechanisms underlying their community assembly. Our findings not only fill gaps in the existing literature on benthic macroinvertebrates in this region but also provide essential baseline data for biodiversity assessment and ecological monitoring in the Jinsha River basin. The results indicate that deterministic processes, such as environmental filtering, are the primary drivers of macroinvertebrate community assembly. This highlights the importance of prioritizing environmental factors in biodiversity conservation strategies.

In addition, this study was limited to a single sampling event. Continuous seasonal monitoring would provide a clearer understanding of macroinvertebrate community diversity patterns and their maintenance mechanisms. Therefore, future macroinvertebrate studies should consider incorporating data from multiple seasons.

## Author Contributions

**Xiaopeng Tang:** conceptualization (equal), data curation (equal), methodology (equal), software (equal), writing – original draft (equal). **Kunyu Shang:** conceptualization (equal), investigation (equal). **Lin Chen:** investigation (equal), methodology (equal). **Chunling Wang:** investigation (equal), validation (equal). **Fubin Zhang:** funding acquisition (equal), investigation (equal), writing – review and editing (equal). **Pengcheng Lin:** funding acquisition (equal), investigation (equal), writing – review and editing (equal).

## Conflicts of Interest

The authors declare no conflicts of interest.

## Supporting information


**Table S1:** Macroinvertebrate traits.

## Data Availability

The data that support the findings of this study are available from the corresponding author upon reasonable request. All data generated or analyzed in this study are contained in the Science Data Bank (https://www.scidb.cn/en/s/mQ7RFr).
