# Supplementary material for: Environmental Filtering Maintains Macroinvertebrate Diversity in the Upper Jinsha River
Source: Ecol Evol. 2025 Oct 16;15(10):e72323. doi: 10.1002/ece3.72323 (PMC12531354; doi:10.1002/ece3.72323)
Supplement: Supplementary file 1 — Table S1: Macroinvertebrate traits. [file ECE3-15-e72323-s001.docx]

**Table S1**

Macroinvertebrate traits

| Trait | Trait state | Code |
| --- | --- | --- |
| **Life history** |  |  |
| Voltinism | Semivoltine (< 1 generation/year) | Volt1 |
|  | Univoltine (1 generation/year) | Volt2 |
|  | Bi- or multivoltine (> 1 generation/year) | Volt3 |
| **Mobility** |  |  |
| Occurrence in drift | Rare (catastrophic only) | Drif1 |
|  | Common (typically observed) | Drif2 |
|  | Abundant (dominant in drift samples) | Drif3 |
| Swimming ability | None | Swim1 |
|  | Weak | Swim2 |
|  | Strong | Swim3 |
| **Morphology** |  |  |
| Attachment | None (free-ranging) | Atch1 |
|  | Some (sessile, sedentary) | Atch2 |
|  | Both | Atch3 |
| Armoring | None (soft-bodied forms) | Armr1 |
|  | Poor (heavily sclerotized) | Armr2 |
|  | Good (e.g., some cased caddisflies) | Armr3 |
| Shape | Streamlined (flat, fusiform) | Shpe1 |
|  | Not streamlined (cylindrical, round, or bluff) | Shpe2 |
| Size at maturity | Small (< 9 mm) | Size1 |
|  | Medium (9-16 mm) | Size2 |
|  | Large (> 16 mm) | Size3 |
| **Ecology**  Rheophily | Depositional only  Depositional and erosional  Erosional | Rheo1  Rheo2  Rheo3 |
| Habit | Burrow | Habi1 |
|  | Climb | Habi2 |
|  | Sprawl | Habi3 |
|  | Cling | Habi4 |
|  | Swim | Habi5 |
| Trophic groups | Skate  Collector-gatherer  Collector-filterer  Herbivore (scraper, piercer, and shedder)  Predator (piercer and engulfer)  Shredder (detritivore) | Habi6  Trop1  Trop2  Trop3  Trop4  Trop5 |
